# Supplementary material for: 1000 years of population, warfare, and climate change in pre-Columbian societies of the Central Andes
Source: PLoS One. 2023 Nov 30;18(11):e0278730. doi: 10.1371/journal.pone.0278730 (PMC10688747; doi:10.1371/journal.pone.0278730)
Supplement: S1 Fig — Observed SPD for normalized (a-b) and unnormalized (c-d) radiocarbon dates against 95% Monte-Carlo simulated envelopes (gray areas) from fitted logistic models of population growth for the northern and southern sociocultural areas. Purple (green) vertical bars indicate positive (negative) deviations from the simulated envelope. (DOCX) [file pone.0278730.s001.docx]

Supplementary Materials for

**1000 years of population, warfare, and climate change in pre-Columbian societies of the Central Andes**

Mauricio Lima, Eugenia M. Gayó, Andone Gurruchaga, Sergio A. Estay, Calogero M. Santoro

*Corresponding author, Mauricio Lima: mlima[@bio.puc.cl](mailto:xxxxx@xxxx.xxx)

**This PDF file includes:**

Fig. S1

Fig. S1. Observed SPD for normalized (a-b) and unnormalized (c-d) radiocarbon dates against 95% Monte-Carlo simulated envelopes (gray areas) from fitted logistic models of population growth for the northern and southern sociocultural areas. Purple (green) vertical bars indicate positive (negative) deviations from the simulated envelope.
