# Supplementary material for: 1000 years of population, warfare, and climate change in pre-Columbian societies of the Central Andes
Source: PLoS One. 2023 Nov 30;18(11):e0278730. doi: 10.1371/journal.pone.0278730 (PMC10688747; doi:10.1371/journal.pone.0278730)
Supplement: S2 Table — Parameter values are given in the columns of the table. The best models were selected considering the Bayesian Akaike Information Criteria (BIC) and the coefficient of prediction (σ2) of the simulated total trajectory predictions initiated with the first observed value of the time series. R2 is the coefficient of determination (% of explained variance) of the fitted models. (DOCX) [file pone.0278730.s003.docx]

Supplementary Materials for

**1000 years of population, warfare, and climate change in pre-Columbian societies of the Central Andes**

Mauricio Lima, Eugenia M. Gayó, Andone Gurruchaga, Sergio A. Estay, Calogero M. Santoro

*Corresponding author, Mauricio Lima: mlima[@bio.puc.cl](mailto:xxxxx@xxxx.xxx)

**This PDF file includes:**

Table S2

Table S2.

**Population models (Eqs. 1-5) fitted to the time series data from the southern sociocultural area**. Parameter values are given in the columns of the table. The best models were selected considering the Bayesian Akaike Information Criteria (*BIC)* and the coefficient of prediction (σ^2^) of the simulated total trajectory predictions initiated with the first observed value of the time series. *R^2^* is the coefficient of determination (% of explained variance) of the fitted models.

| **Population models** |  |  | **Climate** | **Warfare** |  |  |  |  |
| --- | --- | --- | --- | --- | --- | --- | --- | --- |
| Normalized | r_m_ | K | α | β | γ | BIC | R^2^ | σ^2^ |
| Climate | 0.075 | -0.0018 | 0.00014 |  |  | -85.76 | 0.33 | 0.67 |
| **Warfare** | **0.16** | **0.006** |  | **-0.078^*^** |  | **-109.63** | **0.63** | **0.87** |
| **Warfare lateral effect** | **0.087** | **0.003** |  |  | **-0.0008^*^** | **-108.54** | **0.62** | **0.75** |
| Climate and Warfare | 0.16 | 0.004 | 0.00008 | -0.076^*^ |  | -106.04 | 0.63 | 0.90 |
| Climate and Warfare | 0.098 | 0.003 | -0.00003 |  | -0.00075^*^ | -106.75 | 0.64 | 0.85 |
| **Unnormalized** |  |  |  |  |  |  |  |  |
| Climate | 0.066 | -0.0022 | 0.00016 |  |  | -76.07 | 0.30 | 0.52 |
| **Warfare** | **0.16** | **0.011** |  | **-0.091^*^** |  | **-103.72** | **0.65** | **0.87** |
| **Warfare lateral effect** | **0.075** | **0.003** |  |  | **-0.0009^*^** | **-104.81** | **0.66** | **0.69** |
| Climate and Warfare | 0.16 | 0.0006 | 0.0004 | -0.087^*^ |  | -100.44 | 0.65 | 0.90 |
| Climate and Warfare | 0.087 | 0.0032 | -0.00002 |  | -0.0008^*^ | -102.26 | 0.67 | 0.73 |
| **Warfare models** |  |  |  |  |  |  |  |  |
| Normalized | μ | λ | ψ |  |  | BIC | R^2^ | σ^2^ |
| Population | -0.059 | 99.97^*^ |  |  |  | -199.94 | 0.84 | 0.96 |
| Population + Climate | 0.002 | 75.81^*^ | -0.0015^*^ |  |  | -205.62 | 0.88 | 0.99 |
| **Population/Climate** | **-0.038** | **1699^*^** |  |  |  | **-230.29** | **0.93** | **0.99** |
| **Unnormalized** |  |  |  |  |  |  |  |  |
| Population | -0.055 | 95.08^*^ |  |  |  | -199.84 | 0.84 | 0.92 |
| Population + Climate | 0.011 | 70.66^*^ | -0.0016^*^ |  |  | -209.06 | 0.89 | 0.98 |
| **Population/Climate** | **-0.037** | **1663^*^** |  |  |  | **-236.72** | **0.94** | **0.99** |
