# Supplementary material for: 1000 years of population, warfare, and climate change in pre-Columbian societies of the Central Andes
Source: PLoS One. 2023 Nov 30;18(11):e0278730. doi: 10.1371/journal.pone.0278730 (PMC10688747; doi:10.1371/journal.pone.0278730)
Supplement: S1 Data — The columns are: the calibrated years before present (calBP), the calendar years (acdc), the normalized and non-normalized Summed Probability Distributions (SPDn and SPDnn), the respective per capita growth rates (Rn and Rnn), the lithic concentrations (Lith%), the warfare intensity (War) and the warfare growth rates (Rwar). (DOCX) [file pone.0278730.s004.docx]

Supplementary Materials for

**1000 years of population, warfare, and climate change in pre-Columbian societies of the Central Andes**

Mauricio Lima, Eugenia M. Gayó, Andone Gurruchaga, Sergio A. Estay, Calogero M. Santoro

*Corresponding author, Mauricio Lima: mlima[@bio.puc.cl](mailto:xxxxx@xxxx.xxx)

**This PDF file includes:**

Data S1

Data S1. (separate file)

Time series data used for fitting and simulating the population dynamic models (Eqs. 1-5) from the northern sociocultural area. The columns are: the calibrated years before present (calBP), the calendar years (acdc), the normalized and non-normalized Summed Probability Distributions (SPDn and SPDnn), the respective per capita growth rates (Rn and Rnn), the lithic concentrations (Lith%), the warfare intensity (War) and the warfare growth rates (Rwar)..

| **calBP** | **acdc** | **SPDn** | **Rn** | **SPDnn** | **Rnn** | **Lith%** | **War** | **Rwar** |
| --- | --- | --- | --- | --- | --- | --- | --- | --- |
| 1950 | -1 | 0.00003 | 0.08761 | 0.00004 | 0.09149 | 38.688 | 2.9374 | -0.0306 |
| 1925 | 25 | 0.00003 | 0.35980 | 0.00004 | 0.36901 | 37.504 | 2.8488 | -0.0315 |
| 1900 | 50 | 0.00005 | 0.25903 | 0.00006 | 0.26496 | 36.938 | 2.7603 | -0.0310 |
| 1875 | 75 | 0.00006 | 0.58579 | 0.00007 | 0.59020 | 37.246 | 2.6761 | -0.0293 |
| 1850 | 100 | 0.00011 | 0.37179 | 0.00013 | 0.37320 | 38.343 | 2.5987 | -0.0268 |
| 1825 | 125 | 0.00016 | 0.15332 | 0.00019 | 0.15422 | 39.738 | 2.5300 | -0.0236 |
| 1800 | 150 | 0.00018 | 0.21538 | 0.00022 | 0.21383 | 40.655 | 2.4709 | -0.0200 |
| 1775 | 175 | 0.00023 | 0.07319 | 0.00028 | 0.06801 | 40.497 | 2.4219 | -0.0162 |
| 1750 | 200 | 0.00025 | 0.05837 | 0.00030 | 0.04891 | 39.185 | 2.3829 | -0.0123 |
| 1725 | 225 | 0.00026 | 0.09583 | 0.00031 | 0.08814 | 37.095 | 2.3537 | -0.0085 |
| 1700 | 250 | 0.00029 | 0.02956 | 0.00034 | 0.02403 | 34.874 | 2.3337 | -0.0048 |
| 1675 | 275 | 0.00030 | 0.00521 | 0.00035 | 0.00046 | 33.255 | 2.3226 | -0.0012 |
| 1650 | 300 | 0.00030 | 0.01581 | 0.00035 | 0.01344 | 32.760 | 2.3197 | 0.0021 |
| 1625 | 325 | 0.00030 | 0.09548 | 0.00035 | 0.09266 | 33.474 | 2.3247 | 0.0053 |
| 1600 | 350 | 0.00033 | 0.22545 | 0.00039 | 0.23126 | 35.116 | 2.3371 | 0.0082 |
| 1575 | 375 | 0.00042 | 0.44136 | 0.00049 | 0.45920 | 37.108 | 2.3563 | 0.0109 |
| 1550 | 400 | 0.00065 | 0.30295 | 0.00077 | 0.31245 | 38.772 | 2.3822 | 0.0133 |
| 1525 | 425 | 0.00088 | 0.22522 | 0.00106 | 0.22825 | 39.649 | 2.4140 | 0.0153 |
| 1500 | 450 | 0.00110 | 0.13828 | 0.00133 | 0.13942 | 39.758 | 2.4512 | 0.0168 |
| 1475 | 475 | 0.00126 | 0.04671 | 0.00153 | 0.00671 | 39.331 | 2.4928 | 0.0178 |
| 1450 | 500 | 0.00132 | 0.06319 | 0.00154 | 0.00224 | 38.427 | 2.5377 | 0.0181 |
| 1425 | 525 | 0.00141 | 0.11821 | 0.00154 | 0.01356 | 37.217 | 2.5841 | 0.0175 |
| 1400 | 550 | 0.00159 | 0.17652 | 0.00156 | 0.04230 | 36.008 | 2.6297 | 0.0161 |
| 1375 | 575 | 0.00189 | 0.14795 | 0.00163 | 0.05050 | 35.110 | 2.6723 | 0.0141 |
| 1350 | 600 | 0.00220 | 0.12696 | 0.00171 | 0.07221 | 34.715 | 2.7101 | 0.0117 |
| 1325 | 625 | 0.00249 | 0.02359 | 0.00184 | 0.07014 | 34.808 | 2.7419 | 0.0090 |
| 1300 | 650 | 0.00255 | -0.03668 | 0.00197 | 0.04543 | 35.021 | 2.7667 | 0.0062 |
| 1275 | 675 | 0.00246 | -0.04622 | 0.00207 | 0.03991 | 34.676 | 2.7839 | 0.0033 |
| 1250 | 700 | 0.00235 | -0.04082 | 0.00215 | 0.01493 | 33.205 | 2.7931 | 0.0003 |
| 1225 | 725 | 0.00226 | -0.14092 | 0.00218 | -0.1127 | 30.728 | 2.7940 | -0.0027 |
| 1200 | 750 | 0.00196 | -0.12166 | 0.00195 | -0.0988 | 28.051 | 2.7864 | -0.0058 |
| 1175 | 775 | 0.00173 | -0.14070 | 0.00177 | -0.0989 | 25.828 | 2.7704 | -0.0089 |
| 1150 | 800 | 0.00151 | -0.23539 | 0.00160 | -0.1376 | 24.205 | 2.7459 | -0.0119 |
| 1125 | 825 | 0.00119 | -0.08201 | 0.00139 | -0.0821 | 22.865 | 2.7134 | -0.0149 |
| 1100 | 850 | 0.00110 | -0.25679 | 0.00128 | -0.2552 | 21.488 | 2.6733 | -0.0176 |
| 1075 | 875 | 0.00085 | -0.28293 | 0.00100 | -0.2792 | 20.145 | 2.6266 | -0.0200 |
| 1050 | 900 | 0.00064 | -0.34403 | 0.00075 | -0.3479 | 19.096 | 2.5746 | -0.0217 |
| 1025 | 925 | 0.00045 | -0.34461 | 0.00053 | -0.3526 | 18.500 | 2.5194 | -0.0224 |
