# Supplementary material for: 1000 years of population, warfare, and climate change in pre-Columbian societies of the Central Andes
Source: PLoS One. 2023 Nov 30;18(11):e0278730. doi: 10.1371/journal.pone.0278730 (PMC10688747; doi:10.1371/journal.pone.0278730)
Supplement: S2 Data — The columns are: the calibrated years before present (calBP), the calendar years (acdc), the normalized and non-normalized Summed Probability Distributions (SPDn and SPDnn), the respective per capita growth rates (Rn and Rnn), the lithic concentrations (Lith%), the warfare intensity (War) and the warfare growth rates (Rwar). (DOCX) [file pone.0278730.s005.docx]

Supplementary Materials for

**1000 years of population, warfare, and climate change in pre-Columbian societies of the Central Andes**

Mauricio Lima, Eugenia M. Gayó, Andone Gurruchaga, Sergio A. Estay, Calogero M. Santoro

*Corresponding author, Mauricio Lima: mlima[@bio.puc.cl](mailto:xxxxx@xxxx.xxx)

**This PDF file includes:**

Data S2

Data S2. Time series data used for fitting and simulating the population dynamic models (Eqs. 1-5) from the southern sociocultural area. The columns are: the calibrated years before present (calBP), the calendar years (acdc), the normalized and non-normalized Summed Probability Distributions (SPDn and SPDnn), the respective per capita growth rates (Rn and Rnn), the lithic concentrations (Lith%), the warfare intensity (War) and the warfare growth rates (Rwar).

| calBP | acdc | SPDn | Rn | SPDnn | Rnn | Lith% | War | Rwar |
| --- | --- | --- | --- | --- | --- | --- | --- | --- |
| 1650 | 300 | 0.0003 | 0.0095 | 0.00034 | 0.00691 | 35.471 | 1.0686 | -0.0329 |
| 1625 | 325 | 0.0003 | 0.0170 | 0.00035 | 0.01118 | 35.704 | 1.0339 | -0.0272 |
| 1600 | 350 | 0.0003 | 0.0429 | 0.00035 | 0.03404 | 36.245 | 1.0062 | -0.0219 |
| 1575 | 375 | 0.0003 | 0.0470 | 0.00036 | 0.03283 | 36.929 | 0.9844 | -0.0175 |
| 1550 | 400 | 0.0003 | 0.0358 | 0.00038 | 0.02419 | 37.562 | 0.9673 | -0.0139 |
| 1525 | 425 | 0.0004 | 0.0388 | 0.00038 | 0.02793 | 37.991 | 0.9540 | -0.0111 |
| 1500 | 450 | 0.0004 | 0.0299 | 0.00040 | 0.02185 | 38.169 | 0.9435 | -0.0091 |
| 1475 | 475 | 0.0004 | 0.0574 | 0.00040 | 0.04086 | 38.110 | 0.9350 | -0.0077 |
| 1450 | 500 | 0.0004 | 0.0789 | 0.00042 | 0.05545 | 37.819 | 0.9277 | -0.0068 |
| 1425 | 525 | 0.0004 | 0.1153 | 0.00044 | 0.06628 | 37.334 | 0.9214 | -0.0062 |
| 1400 | 550 | 0.0005 | 0.1692 | 0.00048 | 0.08748 | 36.731 | 0.9157 | -0.0056 |
| 1375 | 575 | 0.0006 | 0.1656 | 0.00052 | 0.08431 | 36.081 | 0.9107 | -0.0046 |
| 1350 | 600 | 0.0007 | 0.2151 | 0.00056 | 0.15925 | 35.428 | 0.9064 | -0.0032 |
| 1325 | 625 | 0.0008 | 0.1372 | 0.00066 | 0.19683 | 34.760 | 0.9036 | -0.0008 |
| 1300 | 650 | 0.0010 | 0.0852 | 0.00081 | 0.16002 | 33.995 | 0.9029 | 0.0027 |
| 1275 | 675 | 0.0011 | 0.0491 | 0.00095 | 0.11650 | 32.989 | 0.9054 | 0.0077 |
| 1250 | 700 | 0.0011 | -0.0090 | 0.00106 | 0.04189 | 31.633 | 0.9124 | 0.0143 |
| 1225 | 725 | 0.0011 | 0.0568 | 0.00111 | 0.10305 | 29.972 | 0.9255 | 0.0226 |
| 1200 | 750 | 0.0012 | 0.0435 | 0.00123 | 0.07382 | 28.197 | 0.9467 | 0.0325 |
| 1175 | 775 | 0.0012 | 0.0373 | 0.00132 | 0.07003 | 26.483 | 0.9780 | 0.0437 |
| 1150 | 800 | 0.0013 | 0.0437 | 0.00142 | 0.08851 | 24.907 | 1.0216 | 0.0555 |
| 1125 | 825 | 0.0013 | 0.0088 | 0.00155 | -0.00364 | 23.450 | 1.0800 | 0.0673 |
| 1100 | 850 | 0.0013 | 0.0255 | 0.00154 | -0.01572 | 22.084 | 1.1552 | 0.0783 |
| 1075 | 875 | 0.0014 | 0.0224 | 0.00152 | -0.00045 | 20.845 | 1.2492 | 0.0877 |
| 1050 | 900 | 0.0014 | 0.0119 | 0.00152 | -0.02158 | 19.799 | 1.3637 | 0.0948 |
| 1025 | 925 | 0.0014 | 0.0004 | 0.00149 | -0.04304 | 18.982 | 1.4993 | 0.0993 |
| 1000 | 950 | 0.0014 | -0.0138 | 0.00142 | -0.04194 | 18.375 | 1.6558 | 0.1008 |
| 975 | 975 | 0.0014 | -0.0613 | 0.00137 | -0.09166 | 17.926 | 1.8314 | 0.0992 |
| 950 | 1000 | 0.0013 | -0.0795 | 0.00125 | -0.09642 | 17.594 | 2.0223 | 0.0943 |
| 925 | 1025 | 0.0012 | -0.0798 | 0.00113 | -0.07081 | 17.359 | 2.2223 | 0.0863 |
| 900 | 1050 | 0.0011 | -0.0756 | 0.00105 | -0.04461 | 17.215 | 2.4226 | 0.0760 |
| 875 | 1075 | 0.0010 | -0.0112 | 0.00101 | -0.00441 | 17.179 | 2.6138 | 0.0650 |
| 850 | 1100 | 0.0010 | -0.0002 | 0.00100 | -0.00680 | 17.277 | 2.7892 | 0.0540 |
| 825 | 1125 | 0.0010 | -0.0243 | 0.00100 | -0.03742 | 17.535 | 2.9441 | 0.0436 |
| 800 | 1150 | 0.0010 | -0.0517 | 0.00096 | -0.07365 | 18.007 | 3.0754 | 0.0338 |
| 775 | 1175 | 0.0010 | -0.1246 | 0.00089 | -0.16630 | 18.733 | 3.1812 | 0.0246 |
| 750 | 1200 | 0.0008 | -0.1322 | 0.00076 | -0.18269 | 19.688 | 3.2604 | 0.0158 |
| 725 | 1225 | 0.0007 | -0.2375 | 0.00063 | -0.28018 | 20.761 | 3.3121 | 0.0072 |
| 700 | 1250 | 0.0006 | -0.2819 | 0.00048 | -0.32134 | 21.826 | 3.3361 | -0.0013 |
